# Impact of Parental Mental Health and Help-Seeking on Adolescents’ Suicidal Ideations and Help-Seeking Behaviors

**DOI:** 10.3390/ijerph20156538

**Published:** 2023-08-07

**Authors:** Jaehyun Han, Joung-Sook Ahn, Min-Hyuk Kim, Sei-Jin Chang, Jong-Koo Kim, Seongho Min

**Affiliations:** 1 Department of Psychiatry, Yonsei University Wonju College of Medicine, Wonju 26426, Republic of Korea; jhan0805@yonsei.ac.kr (J.H.); jsahn@yonsei.ac.kr (J.-S.A.); mhkim09@yonsei.ac.kr (M.-H.K.); 2 Department of Preventive Medicine, Yonsei University Wonju College of Medicine, Wonju 26426, Republic of Korea; chang0343@yonsei.ac.kr; 3 Department of Family Medicine, Yonsei University Wonju College of Medicine, Wonju 26426, Republic of Korea; kimjk214@yonsei.kr

**Keywords:** suicidal ideation, parent, maternal, adolescent, help-seeking behavior

## Abstract

This study aimed to evaluate the impact of parental mental health on adolescent offspring. Data regarding 6512 families from the 2010–2021 Korean National Health and Nutrition Examination Survey were analyzed; among them, 428 were placed in the suicidal ideation (SI) group and 421 were placed in the matched control (MC) group. This number was selected for the use of the propensity score matching method. The findings highlighted significant associations between parental mental health and adolescent suicidal ideation, with mothers in the SI group having higher odds of Diagnosed Depression (OR 2.109, 1.023–4.350 95% CI), Depressive Mood (OR 2.155, 1.224–3.793 95% CI), and Suicidal Ideation (OR 2.532, 1.322–4.851 95% CI) compared to the MC group. Regarding the fathers, paternal Suicidal Ideation (OR 4.295, 1.747–10.599 95% CI) was the only significant factor for adolescent suicidal ideation. In contrast, maternal depressive symptoms and help-seeking behavior significantly impacted adolescent help-seeking; Maternal Depressive Mood increased with adolescent Help-Seeking (OR 4.486, 1.312–15.340 95% CI) while Maternal Suicidal Ideation reduced the probability of Help-Seeking in the SI group (OR 0.15, 0.031–0.721, 95% CI). Chronic and severe depressive symptoms in mothers could make adolescents less likely to seek help for their suicidal ideations. Therefore, clinicians working with adolescents should prioritize a family-oriented approach.

## 1. Introduction

Adolescent suicide is a complex and critical issue that has observably varied across populations and regions globally. According to the World Health Organization, suicide is one of the leading causes of death among 15–19-year-olds worldwide, with it being responsible for approximately 800,000 deaths annually [1]. Although adolescent suicide rates in high-income countries have seen a decline, it remains a pressing issue in many countries, including the United States and the United Kingdom, where 10.9 and 6.4 per 100,000 people, respectively, died by suicide in 2021 [2,3].

South Korea has been particularly concerned with the adolescent suicide rate in recent years, with approximately 7.9 to 8.5 per 100,000 people committing suicide from 2010 to 2019. Although the highest rate was recorded in 2015 (8.5 per 100,000 people), the suicide rate has remained relatively constant [4]. Unique features of Korea, including a highly competitive educational system that exacerbates academic pressures, cultural stigmas that discourage help-seeking, and a high suicide rate among adults, could lead to the normalization of suicidal behavior and contribute to adolescent suicide [5,6].

Adolescent suicide is a complex issue influenced by multiple factors, such as individual, social, and environmental factors, as well as mental health problems. Among these components, the parental element is one of the greatest contributors to offspring suicidal behavior [7]. The genetic transmission of mental health conditions, the impact of parental mental illness on family functioning, and the quality of the parent–child relationship can increase the risk of suicidal behavior in adolescents [8]. Family conflict, parental divorce, and abuse or neglect also play roles in increasing these odds [9]. Therefore, understanding the impact of parental factors on children’s mental health is crucial for promoting better mental health outcomes among young people. Parents can play a vital role in preventing adolescent suicide by advocating for healthy family relationships, seeking the appropriate help for their offspring’s mental health problems, and reducing stressors in the family environment [10].

In clinical settings, suicidal ideation is considered an emergency requiring urgent intervention. According to research on the ideation-to-action framework, not all suicidal ideation leads to suicide attempts and the risk factors for suicidal ideation should be distinguished from those for suicide attempts [11]. However, suicidal ideation can still be seen as a sign that an individual’s mental health problems have seriously worsened. Therefore, identifying individuals with suicidal ideation is crucial in clinical situations; it is not just for the purpose of preventing suicide attempts [12]. Furthermore, despite the urgency of suicidal ideation, not everyone who experiences it will seek help. Hence, along with research on transitioning from ideation to attempting suicide, there is a need for research on factors that lead individuals from suicidal ideation to seeking help.

Help-seeking behavior is related to how individuals recognize their health issues and take action to seek appropriate support. This behavior ultimately aims to improve their mental health problems and restore their well-being. Thus, to ensure an improvement in the mental health of adolescents, it is essential to understand their help-seeking behaviors [13], especially regarding the impact of positive parental help-seeking behavior. Parents modeling positive help-seeking behaviors provide support, identify issues early, reduce stigma, normalize help-seeking, and remove barriers to care; this can promote positive help-seeking behaviors among their children and encourage them to seek assistance when needed [14,15].

### Aim of This Study

The purpose of this study is to investigate the relationship between parental mental health and help-seeking behavior and adolescent suicidal thoughts and help-seeking behavior. By using a national representative sample, we aim to address the following research questions: (1) Which parental mental health factors have an impact on adolescent suicidal ideation?; (2) What are the parental mental health factors that affect help-seeking behaviors in adolescents with suicidal ideation?; and (3) To what extent do parents’ help-seeking behaviors influence the help-seeking behaviors of adolescents with suicidal ideation? The findings of this study could contribute to a better understanding of the role of parental factors in adolescent mental health and provide insights for developing effective prevention strategies.

## 2. Materials and Methods

### 2.1. Subjects

The data for this study were obtained from the Korean National Health and Nutrition Examination Survey, specifically from the 5th to 8th wave that spanned a total of 12 years (2010–2021). The survey is conducted annually and uses a complex, stratified, multistage, probability-cluster sampling method to obtain nationally representative samples of the Korean population. It is performed by visiting households and its purpose is to collect information on the health and nutrition statuses, health-related behaviors, and healthcare utilization patterns of Koreans. Every year, 4800 households from 192 survey areas are included in the study; the research targets household members aged 1 year and above (approximately 10,000 individuals in total) [16]. This study analyzed some of the 203 questions related to smoking, drinking, physical activity, mental health, and other aspects included in the data regarding health and nutrition statuses.

In this study, the data spanned a 12-year period and included a total population of 90,602 individuals, which represented the larger population of 50,390,432 individuals in South Korea. The total population of adolescents aged 12 to 18 in the survey was 7160. The data for 6460 mothers and 4972 fathers were also found; these data were combined with the data for adolescents to create family unit data, serving as the unit for analysis. Among the 7160 families, 648 individuals without responses for the key variables were excluded from the analysis, leaving a total of 6512 families in the analysis.

### 2.2. Variables

#### 2.2.1. Variables Related to Mental Health

This study analyzed variables evaluating the mental health of adolescents and their parents; depressive mood and suicidal ideation were common factors included in the assessment. Suicidal ideation was assessed through the question “Have you seriously considered suicide in the past 12 months?”. Depressive mood was evaluated through the question “Have you experienced a continuous period of at least two weeks when you felt extremely sad or hopeless, to the point where it significantly affected your daily life in the past 12 months?”. 

Additionally, to assess the presence of mental illness, for adolescents, the assessment included an ADHD diagnosis; meanwhile, for parents, it included diagnoses of depression and alcohol problems. These variables were evaluated based on the question “Have you ever been diagnosed by a doctor?”. 

Each variable was coded based on responses to single questions posed by the visiting surveyors; all of them were binary variables with “yes” or “no” responses.

#### 2.2.2. Health Help-Seeking Behaviors

Help-seeking behaviors were defined as instances where individuals attempted to contact any type of mental health service in the study. The variable was based on responses to the question “Have you received counseling, either in person, by phone, or through the internet, for mental health issues in the past 12 months?”. This question was asked to both the adolescents and their parents.

#### 2.2.3. Demographic Variables

The variables used in this study included demographic factors, such as age, sex, education, household income, number of family members, and family type. The variable related to the education of adolescents was categorized into three groups: middle school or below (≤9th grade), high school (10th–12th grade), and post-high (high school graduates or those attending college). In terms of family composition, the number of families in which parents and their children lived together was 4518. There were 930 single-parent families and 788 families where grandparents lived together. Additionally, there were 17 adolescents living separately from their parents.

#### 2.2.4. Limitation of Single-Item Questionnaires

While a single question may not be the most reliable measure of suicidality, other similar studies have found that it can still be useful as a screening tool [17]. For example, one study used a single-item screening question to identify medical patients that were at risk for suicide and found that it had high sensitivity and specificity in terms of identifying patients with suicidal ideation [18]. Similarly, the National Comorbidity Survey found that a single question demonstrated a good level of sensitivity and specificity in terms of identifying individuals who had attempted suicide [19]. While these studies also acknowledge the limitations of using a single question, they demonstrate the potential usefulness of such a tool, when combined with other measures, in identifying individuals who may require further evaluation or intervention.

This study utilized single-item questionnaires to collect variables for analysis. The questionnaires included specific expressions, such as ‘seriously considered’ when assessing suicidal ideation and ‘where it significantly affected your daily life’ when measuring depressive mood, to ensure content validity. Additionally, explicit timeframes were used, further supporting content validity. 

Furthermore, according to data released by the survey organization, the suicide ideation rates among adults were within a range of −2.0 (−5.4 to 5.3) for males (*p*-value = 0.922) and −2.0 (−5.7 to 1.9) for females (*p*-value = 0.198), from 2013 to 2021. The stability of the suicide ideation rates observed over several years suggested that the single questionnaire approach had test–retest reliability for assessing suicidal ideation.

### 2.3. Statistical Method

#### 2.3.1. Propensity Score Matching

Propensity score matching (PSM) is a useful statistical technique for comparing similar groups of individuals and estimating the impact of a factor on an outcome; it was used here to account for confounding variables that could impact the outcome, namely, age, sex, household income, number of family members, and family type. This method has several steps, including calculating the propensity score for each individual based on observed characteristics, matching individuals in the case group with those in the control group who have similar propensity scores, and analyzing the outcomes of the matched groups to compare them. The advantages of PSM include reducing selection bias, simulating randomization by creating comparable groups, transparency, flexibility, and enabling subgroup analysis. However, PSM also has some limitations, such as being sensitive to matching quality and assuming strong ignorability. In this study, exact matching was used to account for matching quality and only demographic characteristics were matched to combat ignorability [20].

**Figure 1:**
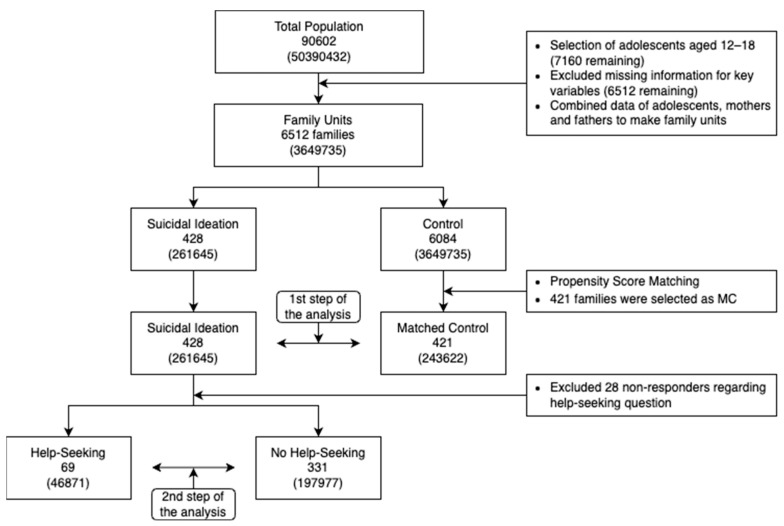
Study Process Flow Chart.

Of all of the subject families, 428 were included in the Suicidal Ideation (SI) group as their adolescents had experienced suicidal ideations within the past 12 months. A matched control (MC) group of 421 families whose adolescents had not experienced suicidal ideations was also included in this study; these families were selected using the PSM method. Within the SI group, 331 families had adolescents who did not seek help for their suicidal ideations (SI group without Help-Seeking, N-HS) while 69 families had adolescents who did seek help (SI group with Help-Seeking, HS). In total, 28 people did not respond to the question about help-seeking. The research flow chart is illustrated in Figure 1.

#### 2.3.2. Analysis of Complex Sample Data

To account for the complex sampling design of the Korean National Health and Nutrition Examination Survey, this study utilized the specialized techniques necessary for analyzing elaborate sample data via the statistical program IBM SPSS Statistics Version 27.0 (IBM Corp., Armonk, NY, USA) and R Version 4.2.3. Such methods are implemented when the data being investigated were collected from an intricately constructed survey, such as stratified, clustered, or multistage, rather than a simple random sample [16]. This provides several benefits, including precision and accuracy, representativeness, variance estimation, handling non-response, flexibility, and subgroup analysis. In this case, weights were used to adjust for the unequal probabilities of selection, non-response, and post-stratification.

#### 2.3.3. Two-Step Analysis

This study involved a two-step analysis designed to examine the relationship between parental mental health and help-seeking behavior and adolescent suicidal thoughts and help-seeking behavior. In the first step, the Suicidal Ideation (SI) group was compared to a matched control (MC) group using the propensity score matching method. In this stage, logistic regression analysis was used to evaluate the impact of mothers’ and fathers’ mental health on adolescents’ suicidal ideations. The odds ratios (ORs) with 95% confidence intervals (CIs) were reported to assess the associations between the variables.

During the subgroup analysis, which was the second step, the SI group was further divided into those who sought help (HS) and those who did not seek help for their suicidal ideation (N-HS); the two groups were ultimately compared. In this step, logistic regression analysis was used to calculate the odds ratios (ORs) with 95% confidence intervals (CIs) to assess the influence of parents’ mental health on adolescents’ help-seeking in comparison to the MC group. Following this, a multivariate regression analysis was performed to calculate the odds ratios between the HS and N-HS groups. This analysis was conducted using a 3-step model in which variables were gradually added.

After these two stages of analysis, an additional analysis was conducted to investigate the interactions between the variables in the second step. The effects package in the statistical program R was utilized to perform the analysis of these interactions; it is commonly used to examine and visualize the interactions between variables in regression models.

## 3. Results

### 3.1. Demographic Characteristics

The demographic characteristics of the total sample, 6512 families with adolescents aged 12–18, are presented in Table 1. Among the adolescents, 3471 were boys (weighted estimates: 2,082,801, 53.2%) and 3041 were girls (weighted estimates: 1,828,578, 46.8%). Significant differences were observed in sex, family type, and maternal marital status when comparing the SI group with the unmatched control group. Within the SI group, the percentage of girls was significantly higher, at 66.2%, than it was in the unmatched control group, at 45.4% (*p*-value < 0.001). Additionally, the proportion of two-parent families in the SI group was significantly lower, at 75.2%, than it was in the control group, at 81.1% (*p*-value = 0.011). Regarding the marital status of mothers in the SI group, the percentage of widowed mothers was higher, at 3.1%, than the control group’s 2.1%; the percentage of mothers who were divorced was also higher, at 10.5%, than the control group’s 5.7% (*p*-value = 0.004). However, while differences were observed in household income and maternal education, they were not statistically significant.

After applying PSM, a matched control group of 421 subjects (243,622 weighted estimates) was selected, consisting of 142 boys (79,174 weighted estimates, 32.5%) and 279 girls (164,448 weighted estimates, 67.5%). In the comparison between the matched control group and the SI group, all of the variables that were previously different from the unmatched control group showed no statistically significant dissimilarities, indicating that the PSM technique effectively controlled for confounding variables.

### 3.2. Comparison between Adolescent Mental Health in SI Group and MC Group

A comparative analysis was performed on the mental health of adolescents in the Suicidal Ideation (SI) group and those in the matched control group (Table S1). The results showed that the odds ratio (OR) for Depressive Mood in the SI group, for the past 12 months, was significantly higher, at 6.759 (4.315–10.586 95% CI), than in the matched control group. Smoking was also found to be significant, with an OR of 3.501 (1.859–6.593 95% CI). In contrast, no significant ORs were observed in the cases of Diagnosed ADHD and Alcohol Use. Furthermore, the OR for Help-Seeking in the SI group was significantly higher than that of the matched control group, with a value of 4.847 (2.547–9.222 95% CI). These findings suggest that adolescents with suicidal ideation have a higher risk of experiencing depressive moods and participating in smoking, as well as having a greater likelihood of seeking help than their matched counterparts that are without suicidal ideation (Figure S1).

### 3.3. Comparison between Parental Mental Health in SI Group and MC Group

This study compared the mental health of the parents in the SI group and the matched control group (Table 2, Figure S2). The results showed that the mothers in the SI group had significantly higher odds of Diagnosed Depression (OR 2.109, 1.023–4.350 95% CI), Depressive Mood in the past 12 months (OR 2.155, 1.224–3.793 95% CI), and Suicidal Ideation (OR 2.532, 1.322–4.851 95% CI) compared to the matched control group. However, when using the 99% confidence interval, Maternal Diagnosed Depression was found not to be significant, suggesting that acute mood symptoms in mothers (Maternal Depressive Mood) may have a greater impact on adolescent suicidal thoughts than chronic stress, such as Maternal Diagnosed Depression.

On the other hand, for fathers, paternal Suicidal Ideation was the only significant factor (OR 4.295, 1.747–10.599 95% CI) in this analysis. These findings suggest that the influence of mothers on adolescent suicidal ideation is different to that of fathers. For adolescent Suicidal Ideation itself, when the 99% CI was applied, neither parents’ Help-Seeking had any significant effect.

### 3.4. Differences between Maternal Mental Health in Both SI Groups and the MC Group

**Figure 2:**
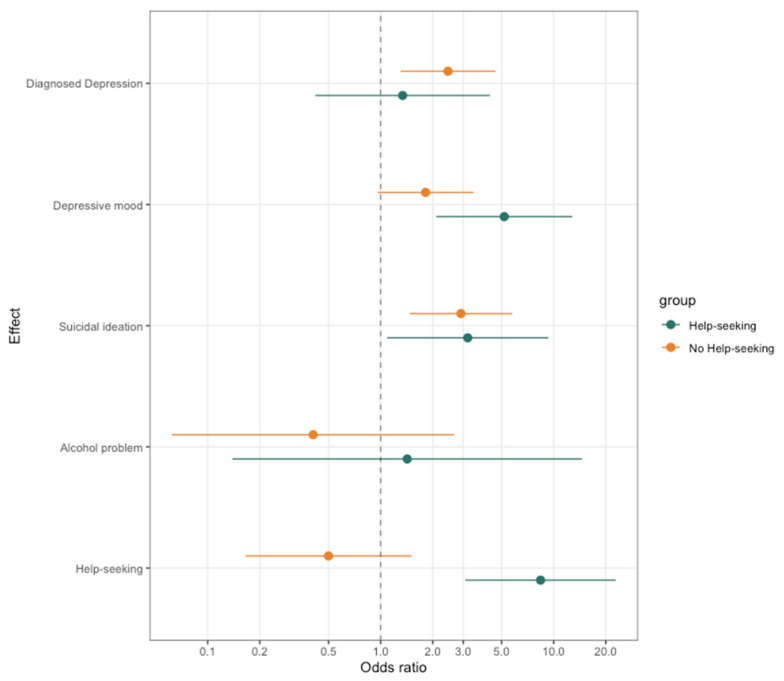
Differences between maternal mental health in the SI groups, with and without Help-Seeking, and the MC group. Odds ratios (ORs) and 95% confidence intervals (CIs) are reported.

The impact of maternal mental health on the SI groups, with and without Help-Seeking, was also examined and compared to the matched control group (Table 3, Figure 2). As the number of observable cases for the fathers was inadequate, an analysis of maternal mental health was carried out instead. The findings revealed that Maternal Diagnosed Depression was only significant in the SI group without Help-Seeking (OR 2.45, 1.304–4.603 95% CI); meanwhile, Maternal Depressive Mood in the past 12 months was significant only in the SI group with Help-Seeking. In the case of Maternal Suicidal Ideation in the past 12 months, both groups showed significant results; however, when the 99% CI was applied, only the SI group with Help-Seeking was found to be significant (OR 2.911, 1.473–5.755 95% CI). This suggests that acute depressive symptoms (Maternal Depressive Mood), rather than a mother’s chronic depression (Maternal Diagnosed Depression), are likely to cause suicidal thoughts that lead adolescents to seek help. However, the results are inconclusive regarding Maternal Suicidal Ideation as it can represent both acute and serious symptoms simultaneously, leading to ambiguity. The significance of the mother’s help-seeking behavior was observed only among adolescents with Suicidal Ideation who sought help (OR 8.399, 3.090–22.831 95% CI) during the analysis.

### 3.5. Results of Multivariate Logistic Regression Analysis regarding Help-Seeking Behavior in the SI Group

Logistic regression analysis was used to perform subgroup analysis on Help-Seeking within the SI group (Table 4); three different models were used in the analysis. In Model 1, a crude logistic regression analysis was performed. In Model 2, a univariate logistic regression analysis was performed after controlling for age, sex, education, household income, number of family members, and family type (Figure S3). In Model 3, a multivariate logistic regression analysis was performed, which included not only the confounding variables controlled for in Model 2 but also the independent variables (Figure S4).

In Model 2, which controlled for confounding variables, Maternal Depressive Mood in the past 12 months was found to be significantly higher in the SI group with Help-Seeking than in the group without Help-Seeking. Even in the multivariate logistic regression analysis (Model 3), Maternal Depressive Mood remained significant (OR 4.486, 1.312–15.340 95% CI). In contrast, interestingly, Maternal Suicidal Ideation was found to have had a significantly negative effect on help-seeking behavior (OR 0.15, 0.031–0.721) in Model 3, unlike in Models 1 and 2. To further evaluate these findings, interaction analysis was required to assess these differences according to the presence or absence of Maternal Suicidal Ideation within the SI group (Table 5).

In terms of maternal Help-Seeking, Model 3 also showed this factor as having a significant effect on Help-Seeking in the SI group (OR 17.495, 1.812–168.877 95% CI). This suggests that maternal Help-Seeking is a crucial factor that affects adolescent help-seeking behavior, even after controlling for other variables.

### 3.6. The Barrier Effect of Maternal Suicidal Ideation on Help-Seeking in Adolescents with Suicidal Ideation

The present study conducted an additional interaction analysis to evaluate the barrier impact of Maternal Suicidal Ideation on help-seeking behavior in adolescents with Suicidal Ideation. In Model 3, the predicted probability was calculated to observe the interaction between Maternal Depressive Mood and Suicidal Ideation (Table 5). The results showed that in subjects who reported Maternal Depressive Mood, Maternal Suicidal Ideation reduced the probability of help-seeking behavior in the SI group, with a decline from about 0.35 to 0.16. However, in subjects without Maternal Depressive Mood, the predicted probability of Maternal Suicidal Ideation was already low, at about 0.23. These findings suggest that Maternal Suicidal Ideation may act as a barrier to help-seeking behavior in adolescents with Suicidal Ideation, particularly when accompanied by Maternal Depressive Mood (Figure S5).

### 3.7. The Impact of Maternal Help-Seeking Behavior on Adolescents’ Help-Seeking Behaviors

The impact of maternal help-seeking behavior on adolescents with Suicidal Ideation was also investigated. Conducting this analysis involved examining the interactions between Maternal Depressive Mood, Suicidal Ideation, and Help-Seeking. As presented in Figure S6, maternal help-seeking behavior increased the predicted probability of Help-Seeking in adolescents with Suicidal Ideation, regardless of the presence or absence of Maternal Depressive Mood and Suicidal Ideation. Consequently, it was suggested that maternal help-seeking behavior plays a significant role in facilitating Help-Seeking among adolescents with Suicidal Ideation, even after controlling for all other variables; these results underscore the importance of parental support and involvement in addressing adolescent mental health issues, particularly when it comes to seeking professional help.

## 4. Discussion

The main purpose of this research was to evaluate the impact of parental mental health on their adolescent offspring’s suicidal ideations and help-seeking behaviors, as well as to investigate how parental help-seeking behaviors influence adolescents. The findings of this study revealed several significant associations that a mother’s Diagnosed Depressive Disorder, Depressive Mood, and Suicidal Ideation in the past 12 months have with Suicidal Ideation in adolescents. Additionally, this study also found that maternal help-seeking behavior has a significant positive effect on adolescent help-seeking behavior, even after controlling for all variables. 

### 4.1. The Impact of Maternal Mental Health on Adolescent Suicidal Ideation

The present study examined the impact of parental mental health on adolescent suicidal ideation and help-seeking behavior, with a focus on Diagnosed Depression, Depressive Mood in the past 12 months, and Suicidal Ideation. Notably, Diagnosed Depression was considered a chronic condition, Depressive Mood an acute condition, and Suicidal Ideation a severe condition. 

The findings indicate that diagnosed depressive disorders in mothers significantly affect adolescent suicidal ideation, possibly due to genetic and environmental factors [21]. Previous research on familial pathways to early-onset suicide attempts has indicated that the offspring of parents that have mood disorders and have attempted suicide are at a higher risk of suicidal behavior than those whose parents are only in one of those categories, suggesting a potential genetic predisposition [9,22]. As well as genetic factors, environmental factors are also capable of playing an important role. Social learning theory proposes that adolescents possibly normalize their depressive behaviors by observing their mothers’ depressive disorders. A mother’s depression can also act as an environmental stressor, leading to emotional neglect and triggering suicidal ideation in adolescents [23]. Moreover, maternal depression can negatively impact parent–child communication, depriving adolescents of emotional support and coping resources [24]. In this study, however, the impact of a mother’s depression diagnosis was not significant in any of the analyses. Chronic effects need to be taken into account; however, additionally, if the mother has a diagnosed depressive disorder and has been receiving treatment, the influence of diagnosed depression on adolescent suicidal ideation may be lessened.

The significant impact of Maternal Depressive Mood in the past 12 months on adolescent suicidal ideation is additionally highlighted by this study. Chronic stress and instability in the home environment, such as in cases of Maternal Diagnosed Depression, can have a pervasive impact on adolescent mental health [25]. Meanwhile, the acute or temporary nature of a Maternal Depressive Mood may act as a specific stressor, triggering suicidal thoughts that require more urgency [26]. Research in the literature has found that poor maternal mental health has a significant effect on adolescent suicidal ideation and suicide attempts, even after controlling for attachment style and family functioning [27]. Furthermore, Maternal Depressive Mood may indicate problems with the mother’s problem-solving, emotion regulation, and communication skills [28], which can negatively impact the mental health of adolescents. Therefore, it is essential to consider the timing and nature of Maternal Depressive Moods.

Compared to a diagnosed depressive disorder or recent depressed mood, Maternal Suicidal Ideation poses the greatest threat to adolescent mental health due to its more severe nature as a stressor. The association between maternal suicidal behavior and offspring suicidal behavior was found to be strongest when the mother had attempted suicide [29]. Although the number of observed cases of maternal suicidal attempts was limited in this community sample, these results centralize the importance of addressing severe mental health problems in mothers, such as suicidal ideation, for the prevention of adolescent suicidal behavior. Further research is needed to investigate this topic. Overall, this study underscores the value of considering the different facets of parental mental health when addressing adolescent suicidal ideation and highlights the need for appropriate interventions.

### 4.2. Gender Differences in the Effects of Parental Mental Health on Children’s Suicidal Ideations

Potential gender differences in the effects of parental mental health on adolescent suicidal ideation were also examined in this study. It was discovered that girls reported suicidal ideation more commonly than boys and that depression and suicidal thoughts were observed more frequently in mothers than in fathers. Moreover, even after controlling for gender, an adolescent’s suicidal ideation was found to be primarily influenced by their mother’s mental health.

There are significant gender differences in regard to suicidal behavior, with men having a higher likelihood of dying by suicide and women being more prone to suicide attempts [30]. In line with this ‘gender paradox’; a meta-analysis of 50 years of research on suicide reported higher rates of suicide completion in men and, in contrast, higher rates of non-suicidal self-injury in women [31]. Similarly, studies of adolescent suicidal ideation have shown that women are more likely to report mental illness and suicidal ideation while men are more likely to report suicidal attempts. Thus, this study, conducted with a community sample, certainly omitted those who had committed suicide and likely omitted those who were experiencing severe mental illness; this suggests that more women than men with suicidal thoughts were included in the sample.

Another gender difference observed in this study was that the impact of maternal mental health on adolescent suicidal ideation was more evident than that of paternal mental health. A study examining familial pathways to early-onset suicide attempts found that fathers’ mental health had a significant impact on their children’s suicidal ideations; however, the effect was still smaller than that of mothers [22]. Another study reported that a mother’s depression and anxiety were associated with adolescent suicidal ideation while a father’s depression and anxiety were not; the study explained that the results were mediated, in part, by the parent–child relationship [32]. Differences in parenting styles, the amount of time spent together, and the gender roles that exist between mothers and fathers could explain the contrast in the influences of maternal and paternal mental health [33,34]. In this study, depressive symptoms were perceived as more visible in mothers than in fathers, resulting in greater disruptions in the family environment and increased stress for adolescents; paternal depressive symptoms may be overlooked or adolescents may perceive them as less severe [35,36]. Highlighting the importance of considering the different effects of maternal and paternal mental health on adolescent suicidal ideation, this study centralizes the need for further research on this topic.

### 4.3. The Impact of Maternal Mental Health on Adolescents’ Help-Seeking Behaviors

This study revealed that experiencing a mother with a depressive mood within the past year had a positive influence on adolescents’ help-seeking behaviors; the mother having diagnosed depression did not have this impact. Moreover, the presence of maternal suicidal ideation was found to reduce adolescents’ help-seeking behaviors. These results highlight the complex nature of the impact of maternal depressive symptoms on adolescents’ help-seeking behaviors.

An abundance of studies examining the impact of maternal mental health on adolescent help-seeking behavior have yielded mixed results [25,37]. For example, one study of African American and Latino adolescents with suicidal ideation found that maternal depression or depressive symptoms can act as barriers to help-seeking behavior [38]. In contrast, other studies have concluded that adolescents with parents or relatives who have a history of mental health problems are more likely to seek professional help for their own mental health issues [39,40]. Two studies have shown that mothers who have personal experiences with mental health diagnoses and treatments, as well as those who have positive perceptions and beliefs about medication, are more likely to seek and accept help for their children’s mental health needs [41,42]. In a single study, it was found that a family history of self-harm was positively associated with seeking professional help independently, within the international sample that was analyzed [43]. The severity of parental mental health and parental experiences of therapy appear to be key factors in determining whether or not parental mental health impacts adolescent help-seeking behavior. 

Given the perspectives of these studies, it could be determined that the impact of previously diagnosed depression in the present study may not be severe enough to significantly impact adolescent help-seeking behavior. It could otherwise be suggested that parents who have experienced treatment may be prone to seeking help for their children’s mental illness [44]. However, it is the case that a mother’s current depressive mood can be felt acutely by adolescents and emotional contagion from the mother’s emotional state can cause adolescents to feel hopeless or isolated [45]. Adolescents are likely to feel that their most intimate caregiver, their mother, is less responsive and aware of them, a circumstance capable of creating communication barriers for adolescents and leading them away from help-seeking behaviors [46,47]. In a longitudinal study about patterns of help-seeking behavior, a poorer parent attachment reduced help-seeking behavior [48]. 

The severity of a mother’s emotional symptoms can also have a notable impact on her child’s help-seeking behavior by encouraging negative beliefs toward mental health services and professionals. A systematic review found that a mother’s severe depression or suicidal ideation can cause a feeling of helplessness in her offspring, with many of them dismissing treatment as useless; this feeling is the most cited barrier to seeking help [49,50]. The impact of these negative perceptions of therapy may be greater if the mother receives treatment, especially as the symptoms often persist, which can further undermine the adolescent’s trust in mental health services [51]. In another study, it was found that higher levels of suicidal ideation paradoxically led to lower rates of help-seeking behavior [52,53,54]. This suggests that in the case of adolescents with parents with severe mental disorders, the adolescent’s level of suicidal ideation is more serious due to their exposure to a more devastating genetic predisposition and set of environmental factors, both of which lead to a reduction in help-seeking behavior. This highlights the need for early detection and intervention in adolescents with severe mental disorders and suicidal ideations, as well as the importance of addressing the barriers to help-seeking behavior when working with this population [55].

### 4.4. The Adolescent’s Help-Seeking Facilitated by the Mother’s Help-Seeking

This study aimed to investigate the impact of a mother’s help-seeking behavior on the help-seeking behaviors of adolescents with suicidal thoughts. The results showed that a mother’s help-seeking behavior has a significant effect on her adolescents’ help-seeking behaviors, independently of other variables. These findings are consistent with previous studies that highlight the importance of evaluating and intervening with parents, particularly mothers, when working with adolescents with suicidal thoughts [56,57,58]. A systematic review of 14 studies also found a significant association between mothers’ help-seeking and their offspring’s help-seeking behaviors [59]. The study indicated that when mothers sought help for their suicidal behavior, their children were more likely to receive help and ultimately had better treatment outcomes. Similarly, according to another recent systematic review study, parental help-seeking behaviors may have a positive impact on the mental health outcomes of children and adolescents. A total of 24 relevant studies were reviewed, with the resulting evidence suggesting that when parents seek help for their children’s mental health concerns, it can lead to improved outcomes, including reduced symptoms of anxiety and depression [50].

The current study’s findings suggested that even after controlling for the degree or severity of the mother’s depression, a mother’s help-seeking behavior had a significant effect on her adolescents’ help-seeking behaviors. These findings emphasize the need for an active therapeutic approach and an evaluation of the main caregivers when proceeding with the therapeutic intervention of adolescents with suicidal thoughts [50]. It is crucial to consider the role of parents, particularly mothers, in facilitating adolescents’ help-seeking behaviors and improving their mental health outcomes [60].

### 4.5. Cultural Difference When Compared to Western Samples

This study analyzed a sample representing the South Korean population; therefore, when generalizing the results, a consideration of cultural aspects is necessary. South Korea has one of the highest suicide rates among developed countries [1]. According to the WHO, the suicide rate in South Korea was approximately 24.6 suicides per 100,000 people in 2016, which was higher than the average global suicide rate of 10.6 suicides per 100,000 people in the same year. Various cultural, social, economic, and individual factors are involved in this phenomenon. Additionally, the tendency to view mental health and mental problems as burdensome, in families and in society, acts as a barrier to help-seeking behavior among individuals with suicidal ideation.

According to the same report from the WHO, the suicide rate among Korean adolescents in 2016 was approximately 7.1 suicides per 100,000 people, which is not as high as the suicide rate among adults but is still higher than the average rate in Western countries. As mentioned in the introduction, this is associated not only with cultural and social factors, similarly to adults, but also with intense academic pressures from a highly competitive education system. The high suicide rate among adults may not only normalize suicidal ideation among adolescents but also hinder their help-seeking behaviors [61]. 

Regarding the method of suicide, among both boys and girls, hanging was the most common method in South Korea, which differs from Western countries. Particularly among adolescents in regions where firearms are more accessible; firearms are the most common method of suicide in the United States and many European countries. Due to differences in accessibility to lethal suicide methods, there may indeed be variations in the rate at which suicidal ideation progresses to actual suicide attempts between Korean and Western adolescents [62].

### 4.6. Limitation

As highlighted by this study’s results, the roles of maternal mental health and help-seeking behavior in promoting better mental health outcomes among adolescents are critical. Regarding the help-seeking behavior of adolescents, the mother’s acute symptoms had a greater influence than the chronic symptoms; meanwhile, the severe symptoms acted as barriers to seeking help. Despite the value of this information, there are several limitations that should be considered when discussing the implications of this study.

The primary limitation of this study is that the variables were obtained from participant responses to a single question, rather than a validated scale, which may have raised concerns regarding both reliability and validity [12]. However, as with other studies, this single question may serve as a reliable screening tool; also, the variables in question were not the only ones considered in this analysis [18]. This study’s use of single-item questionnaires with specific expressions and explicit timeframes helped ensure content validity. Furthermore, the consistent suicidal rate observed in this study over the years suggests that the measurement of suicidal ideation using the single-item questionnaire is reliable over time and provides consistent results. This study’s large sample size and ability to be nationally representative also add to its reliability. 

Another limitation is the limited scope of the variables, which did not include family communication level, peer relationships, and academic stress, all of which may impact adolescent suicidal ideation and help-seeking behavior [63]. To combat this issue, however, this study minimized confounding variables using the PSM method. PSM is a valuable technique for reducing bias, especially when applied to large samples. For example, although single parenthood was found to have a significant effect on adolescent suicidal ideation and help-seeking behavior, the effect was minimized through matching and controlling. Without question, in this particular study, the advantages of using PSM outweighed the potential challenges related to overfitting and the assumptions of unconfoundedness and covariate balance, especially considering the ability to provide a more elaborate analysis with a small number of cases, such as suicidal ideation.

Finally, because this study used only one question addressing suicidal thoughts, it was not possible to distinguish between suicidal ideation and intention, or between non-suicidal self-injury and suicide. Nonetheless, given the screening ability of the question ‘Have you had thoughts about suicide in the past 12 months?’ in the general community, this study’s findings hold important clinical value.

## 5. Conclusions

Based on the significant associations identified between parental mental health and adolescent suicidal ideation and help-seeking behavior, it is crucial that parental mental health is considered an essential factor in identifying and addressing adolescent mental health concerns. Findings evidencing the fact that maternal help-seeking behavior positively impacts adolescent help-seeking behavior are particularly noteworthy, given that maternal depressive symptoms (both chronic and severe) could make adolescents less likely to seek help. Therefore, it is paramount that mothers are encouraged to seek help for their own mental health concerns. Furthermore, clinicians and mental health professionals working with adolescents should prioritize family mental health history and encourage open communication between parents and their children regarding mental health. Overall, this study underscores the need for further research and interventions that promote family-based approaches to improving adolescent mental health outcomes.

## Figures and Tables

**Table 1:** Demographic Characteristics.

			Total			Suicidal Ideation Group			Unmatched Control Group					Matched Control Group				
			*N*	Weighted N	%	*N*	Weighted N	%	*N*	Weighted N	%	X^2^	*p*-Value	*N*	Weighted N	%	X^2^	*p*-Value
			6512	3,911,379		428	261,645		6084	3,649,735				421	243,622			
Adolescent	Age		15.13 (SD 0.28)			15.36 (SD 0.105)			15.11 (SD 0.029)			3.838	0.004	15.34 (SD 0.098)			0.018	0.481
	Sex	Boy	3471	2,082,801	53.2	144	88,450	33.80	3327	1,994,352	54.60	71.113	<0.001	142	79,174	32.50	0.001	0.979
		Girl	3041	1,828,578	46.8	284	173,195	66.20	2757	1,655,383	45.40			279	164,448	67.50		
	School Status	Middle	3509	1,815,439	46.5	215	8990	41.00	3294	39,475	46.80	5.938	0.115	209	104,481	43.20	3.093	0.542
		High	2428	1,696,921	43.4	163	10,791	44.80	2265	40,858	43.30			172	113,045	46.70		
		Post-High	408	287,997	7.4	37	4742	9.60	371	15,924	7.20			28	17,954	7.40		
		Dropout	161	107,942	2.8	13	3742	4.60	148	11,029	2.60			9	6627	2.70		
	ADHD	Yes	84	53,036	1.4	6	3469	1.30	78	49,567	1.40	0.055 *	0.834	5	2838	1.20	0.074	0.786
		No	6422	3,854,652	98.6	422	258,176	98.70	6000	3,596,476	98.60			415	240,148	98.80		
Family	No. of FM	4 or less	4857	2,924,359	74.8	319	190,051	72.90	4538	2,734,308	74.90	0.002 *	0.961	316	183,024	75.10	0.014	0.906
		5 or more	1653	985,180	25.2	108	70,718	27.10	1545	914,461	25.10			105	60,598	24.90		
	Family Type	TP	5306	3,141,442	80.7	329	195,799	75.20	4977	2,945,643	81.10	8.956	0.011	327	186,432	76.50	0.024	0.988
		SP	930	590,202	15.2	70	45,840	17.60	860	544,361	15.00			68	42,574	17.50		
		etc	258	162,873	4.2	27	18,577	7.10	231	144,296	4.00			26	14,617	6.00		
	Income	1Q	713	478,251	12.3	61	45,920	17.80	652	432,331	11.90	6.58	0.087	60	38,160	15.70	0.007	1
		2Q	1628	1,038,550	26.7	101	68,071	26.30	1527	970,479	26.80			101	66,418	27.30		
		3Q	2086	1,219,660	31.4	123	69,305	26.80	1963	1,150,355	31.70			123	66,932	27.50		
		4Q	2036	1,147,222	29.5	137	75,299	29.10	1899	1,071,924	29.60			137	72,112	29.60		
Mother	Education	Primary	152	105,687	3.2	13	10,138	4.90	139	95,548	3.10	7.035	0.071	17	10,384	4.90	1.561	0.668
		Middle	223	146,409	4.4	17	12,867	6.20	206	133,542	4.30			16	9018	4.30		
		High	2772	1,667,826	50.6	192	117,204	56.60	2580	1,550,622	50.20			183	111,626	52.90		
		Post-high	2423	1,375,831	41.7	131	66,983	32.30	2292	1,308,848	42.40			147	79,796	37.80		
	Marital Status	Married	5490	3,2343,590	91.9	330	190,926	86.40	5160	3,043,425	92.20	11.043 *	0.004	350	200,097	90.10	1.852	0.396
		Widowed	114	76,135	2.2	10	6865	3.10	104	69,270	2.10			6	3157	1.40		
		Divorce	305	210,344	5.90	32	23,172	10.50	273	187,172	5.70			27	18,873	8.50		
**Father**	**Education**	Primary	105	74,692	3.20	10	5468	3.80	7	4307	3.10	3.053	0.384	7	4,307	3.10%	3.053	0.384
		Middle	279	169,302	7.10	21	16,230	11.40	13	7272	5.20			13	7,272	5.20%		
		High	1547	928,047	39.10	96	60,997	42.70	95	56,737	40.40			95	56,737	40.40%		
		Post-high	2148	1,199,015	50.60	126	60,229	42.10	139	71,998	51.30			139	71,998	51.30%		
	**Marital** **Status**	Married	4505	2,633,664	96.60	269	153,378	96.40	281	157,084	98.30	1.111	0.574	281	157,084	98.30%	1.111	0.574
		Widowed	21	9,934	0.40	1	420	0.30	2	666	0.40			2	666	0.40%		
		Divorce	125	81,352	3.00	8	5237	3.30	5	2100	1.30			5	2100	1.30%		

Note. FM; family members, TP; two-parent family, SP; single-parent family. * Statistically significant results (*p* < 0.05). A complex sampling analysis was used.

**Table 2:** Comparison of parental mental health between the SI group and MC group.

			Suicidal Ideation Group			Matched Control Group			OR	95% CI	
			N	Weighted N	%	N	Weighted N	%		Lower	Higher
Mother	Diagnosed Depression	N	153	97,170	86.90	249	151,334	93.40	2.109 *	1.023	4.35
		Y	26	14,587	13.10	20	10,771	6.60			
	Depressive Mood	N	238	137,013	78.50	231	129,591	88.70	2.155 *	1.224	3.793
		Y	59	37,454	21.50	28	16,440	11.30			
	Suicidal Ideation	N	251	142,450	81.60	239	134,126	91.80	2.532 *	1.322	4.851
		Y	46	32,016	18.40	20	11,905	8.20			
	Alcohol Use	N	325	190,639	98.70	343	198,933	98.10	0.672	0.165	2.748
		Y	4	2469	1.30	6	3832	1.90			
	Help- Seeking	N	308	178,889	94.60	301	174,144	96.50	1.576	0.645	3.855
		Y	14	10,224	5.40	11	6313	3.50			
Father	Diagnosed Depression	N	100	63,473	97.10	170	100,474	97.40	1.099	0.232	5.215
		Y	5	1886	2.90	4	2717	2.60			
	Depressive Mood	N	186	99,661	86.10	176	92,794	91.50	1.725	0.744	3.996
		Y	22	16,065	13.90	14	8673	8.50			
	Suicidal Ideation	N	180	96,250	83.20	179	96,902	95.50	4.295 *	1.747	10.559
		Y	28	19,476	16.80	11	4566	4.50			
	Alcohol Use	N	241	135,018	97.90	253	138,850	98.90	1.81	0.394	8.316
		Y	6	2842	2.10	3	1615	1.10			
	Help- Seeking	N	223	126,104	98.10	225	124,288	99.80	10.742 *	1.046	110.28
		Y	4	2489	1.90	1	228,354	0.20			

Note. * Statistically significant results (*p* < 0.05). Odds ratio (OR) and 95% confidence intervals (CIs) are reported.

**Table 3:** Differences in maternal mental health in the SI groups, with and without Help-Seeking, compared to the MC group.

	Diagnosed Depression		Depressive Mood		Suicidal Ideation		Alcohol Use		Help-Seeking	
	OR	95% CI	OR	95% CI	OR	95% CI	OR	95% CI	OR	95% CI
MC	1(ref)		1(ref)		1(ref)		1(ref)		1(ref)	
N-HS	2.45 *	1.304–4.603	1.818	0.961–3.439	2.911 *	1.473–5.755	0.407	0.062–2.66	0.499	0.165–1.508
HS	1.339	0.419–4.279	5.179 *	2.095–12.801	3.185 *	1.088–9.329	1.424	0.139–14.59	8.399 *	3.09–22.831

Note. MC; matched control group, N-HS: suicidal ideation group without help-seeking behavior, HS: suicidal ideation group with help-seeking behavior. * Statistically significant results (*p* < 0.05). Odds ratios (ORs) and 95% confidence intervals (CIs) are reported.

**Table 4:** Results of multivariate logistic regression analysis, according to help-seeking behavior in the SI group.

	Model 1			Model 2			Model 3		
	OR	95% CI		OR	95% CI		OR	95% CI	
		Lower	Upper		Lower	Upper		Lower	Upper
**Diagnosed** **Depression**	0.763	0.361	1.612	0.603	0.256	1.422	0.303	0.085	1.077
**Depressive** **Mood**	3.112 *	1.473	6.577	3.552 *	1.612	7.828	4.486 *	1.312	15.34
**Suicidal** **Ideation**	1.184	0.488	2.869	1.078	0.352	3.297	0.150 *	0.031	0.721
**Help-** **Seeking**	16.434 *	5.126	52.688	14.115	3.542 *	56.251	17.495 *	1.812	168.877

Note. Model 1: unadjusted. Model 2: adjusted for variables (age, sex, education, household income, number of family members, and family type). Model 3: adjusted for variables (age, sex, education, household income, number of family members, and family type, the other independent variables; Diagnosed Depression, Depressive Mood, Suicidal Ideation, and Help-Seeking in the mother). * Statistically significant results (*p* < 0.05). Odds ratios and 95% confidence intervals are reported.

**Table 5:** The barrier effect of Maternal Suicidal Ideation on Help-Seeking in adolescents with Suicidal Ideation.

		Maternal Suicidal Ideation					
		No			Yes		
		Effect	95% CI		Effect	95% CI	
			Lower	Upper		Lower	Upper
**Maternal** **Depressive Mood**	No	0.23178138	0.12852761	0.3816575	0.09909794	0.02607699	0.3112460
	Yes	0.34637910	0.15075618	0.6127045	0.16192120	0.05832948	0.3760246

## Data Availability

Publicly available datasets were analyzed in this study. These data can be found here: [https://knhanes.kdca.go.kr/knhanes/main.do] (accessed on 7 May 2023).

## Supplementary Materials

The following supporting information can be downloaded at: https://www.mdpi.com/article/10.3390/ijerph20156538/s1. Table S1: Comparison of Adolescent Mental Health between SI Group and MC Group; Figure S1: Comparison of Adolescent Mental Health between SI Group and MC Group; Figure S2: (a) Comparison of Maternal Mental Health between SI Group and MC Group; (b) Comparison of Maternal Paternal Health between SI Group and MC Group; Figure S3: Logistic Regression Analysis after adjusting Demographic Variables (Model 2); Figure S4: Multivariate Logistic Regression Analysis according to Help-Seeking Behavior in SI Group (Model 3); Figure S5: The Interaction Impact of Maternal Suicidal Ideation on Help-Seeking in Adolescents with Suicidal Ideation; Figure S6: The Impact of Maternal Help-Seeking Behavior on Adolescents’ Help-Seeking Behaviors.
