# Supplementary material for: Impact of Parental Mental Health and Help-Seeking on Adolescents’ Suicidal Ideations and Help-Seeking Behaviors
Source: Int J Environ Res Public Health. 2023 Aug 7;20(15):6538. doi: 10.3390/ijerph20156538 (PMC10419277; doi:10.3390/ijerph20156538)
Supplement: Supplementary file 1 [file ijerph-20-06538-s001.zip › ijerph-2411961-supplementary.pdf]

Table S1. Comparison of Adolescent Mental Health between SI Group and Matched Control

		Suicidal Ideation			Matched control			OR	95% CI	
		N	Weighted N	%	N	Weighted N	%		lower	higher
ADHD	N	422	258176	98.70	415	240148	98.80	1.137	0.309	4.186
	Y	6	3469	1.30	5	2838	1.20			
Depressive Mood	N	255	154353	59.00	385	220670	90.70	6.759*	4.315	10.586
	Y	173	107291	41.00	35	22695	9.30			
Alcohol Use	N	272	154134	74.30	283	163713	76.00	1.094	0.725	1.652
	Y	82	53259	25.70	86	51711	24.00			
Smoking	N	352	206592	85.20	387	221686	95.30	3.501*	1.859	6.593
	Y	49	35907	14.80	18	11006	4.70			
Help-Seeking	N	331	197977	80.90	340	195945	95.30	4.847*	2.547	9.222
	Y	69	46872	19.10	16	957	4.70			

Note. ADHD; attention-deficit hyperactivity disorder *Statistically significant results ($p < 0.05$). Odds ratio (OR) and 95% confidence intervals (CI) are reported.

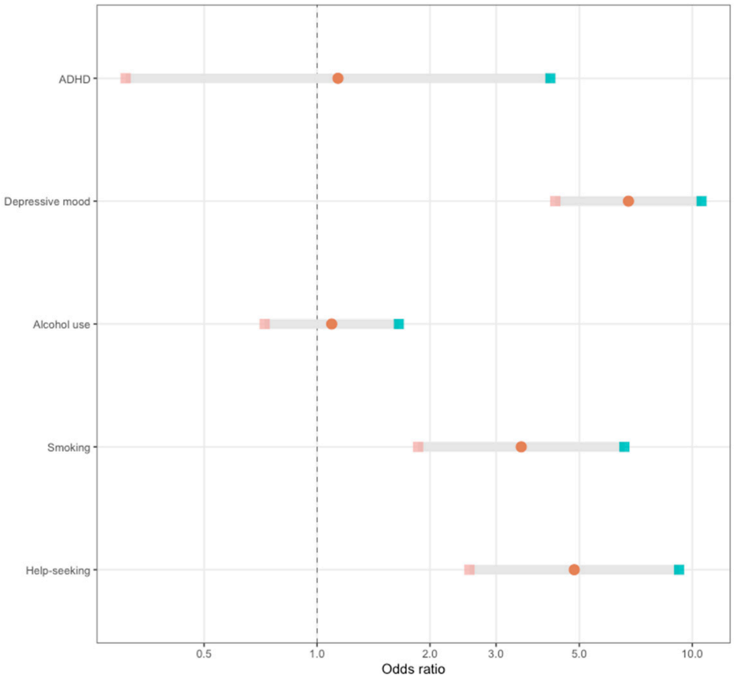

Figure S1. Comparison of Adolescent Mental Health between SI Group and Matched Control. Odds ratio (OR) and 95% confidence intervals (CI) are reported.

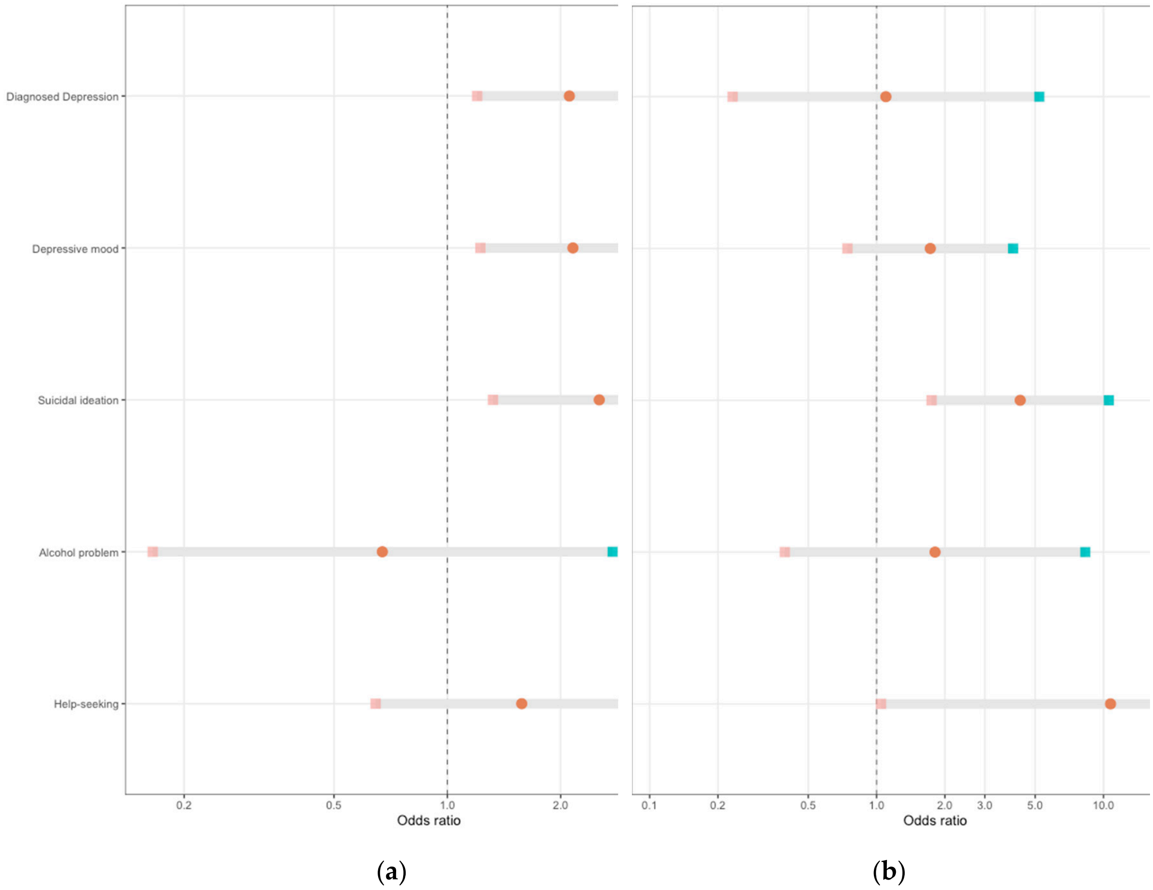

Figure S2. (a) Comparison of Maternal Mental Health between SI Group and Matched Control group; (**b**) Comparison of Maternal Paternal Health between SI Group and Matched Control group. Odds ratio (OR) and 95% confidence intervals (CI) are reported.

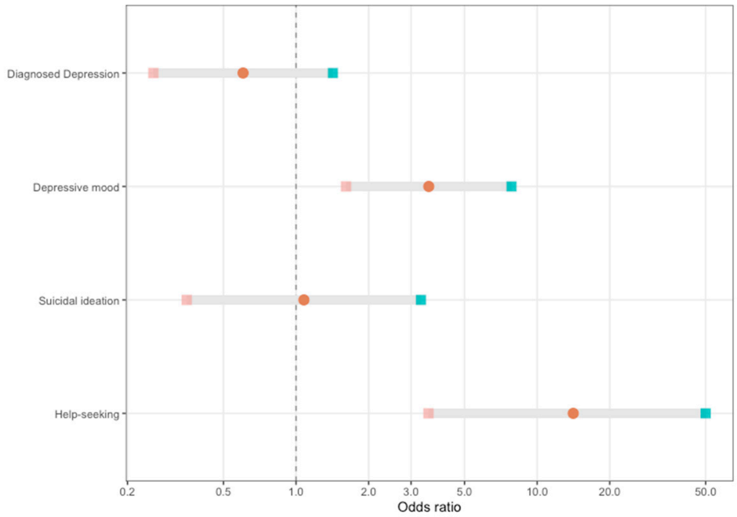

Figure S3 Logistic Regression Analysis after adjusting Demographic Variables (Model 2). Odds ratio (OR) and 95% confidence intervals (CI) are reported.

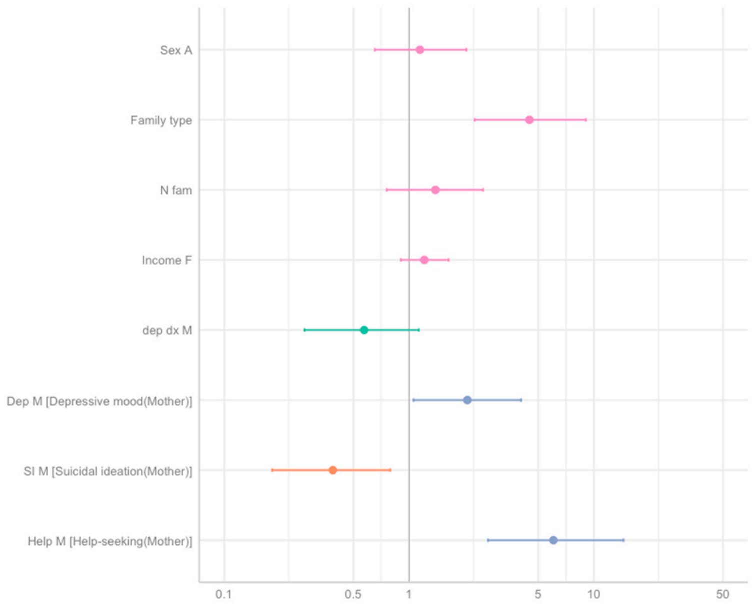

Figure S4 Multivariate Logistic Regression Analysis according to Help-Seeking Behavior in SI group (Model 3). Odds ratio (OR) and 95% confidence intervals (CI) are reported.

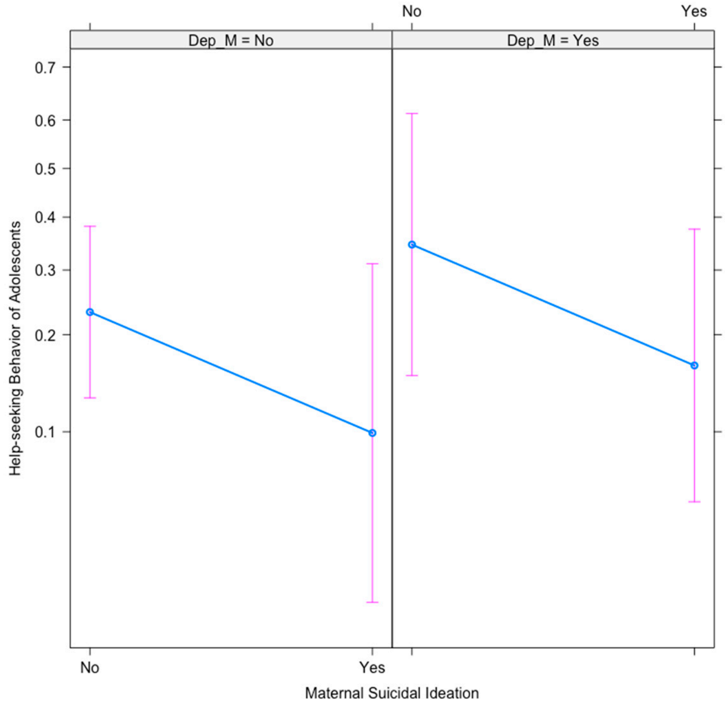

Figure S5 The Interaction Impact of Maternal Suicidal Ideation on Help-Seeking in Adolescent with Suicidal Ideation.

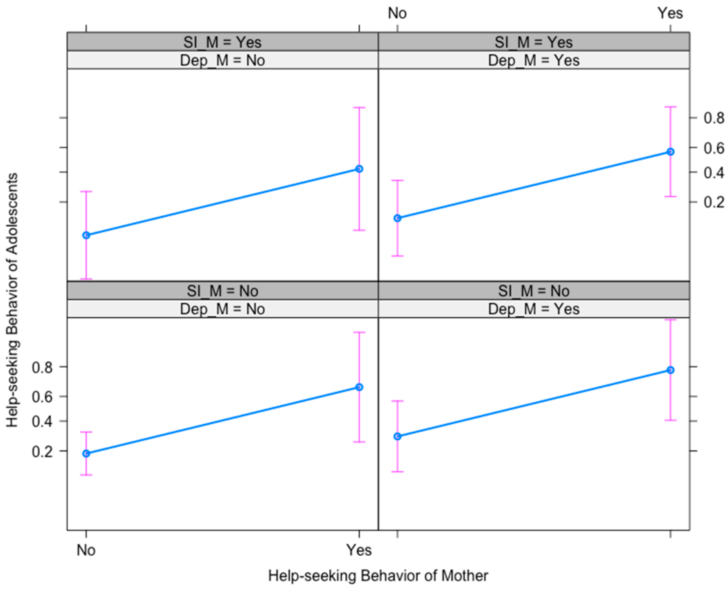

Figure S6 The Impact of Maternal Help-Seeking Behavior on Adolescents'
